# Dietary Habits and Metabolic Health

**DOI:** 10.3390/nu15183975

**Published:** 2023-09-14

**Authors:** Bowen Li, Xue Tang, Guowei Le

**Affiliations:** 1 State Key Laboratory of Food Science and Resources, Jiangnan University, Wuxi 214122, China; tangxue@jiangnan.edu.cn (X.T.); lgw_jiangnan@163.com (G.L.); 2 School of Food Science and Technology, Jiangnan University, Wuxi 214122, China

Dietary habits refer to the long-term dietary patterns and habits that an individual forms and maintains in their daily life. Dietary behavior is an essential and ongoing activity in daily life, which involves internal, external, and conscious activities related to eating. With a deeper understanding of nutritional science, people are increasingly aware of the complex relationship between dietary habits and health outcomes. 

Exploring the variations in nutrient composition in food contributes to the formation of proper dietary habits. Positive dietary behaviors are important strategies for individuals to ensure their health. Eating regular meals, moderating the intake of fats and sugars, consuming an adequate number of fruits and vegetables, paying attention to nutritional labels and calorie content, practicing good hygiene, and choosing foods based on principles of nutrition and health are all considered behaviors that promote physical well-being. However, as the pace of life accelerates, various fast-food products and the rapid development of the ultra-processed food industry have flooded the market. Ultra-processed foods refer to foods that have undergone packaging, processing, and the addition of numerous additives, preservatives, seasonings, and added sugars. These foods often lack a variety of nutrients such as fiber, vitamins, and minerals, while containing excessive amounts of sugar, salt, and unhealthy fats [1]. This leads to nutritional imbalance and can contribute to metabolic health problems such as obesity, diabetes, and cardiovascular disease. In these processed foods, significant changes have occurred in the nutritional composition, including the presence of protein oxidation products, lipid oxidation products, and advanced glycation end products [2]. These products not only stimulate the appetite center, making individuals more prone to excessive intake of these unhealthy components, but they also potentially have a negative impact on the gut microbiota, disrupting the balance of beneficial bacteria and compromising gut health.

In addition to reducing the intake of harmful substances in food, dietary patterns with nutrient interactions and synergistic effects, or specific food combinations, have become popular dietary strategies for the prevention of metabolic diseases. A whole grain diet refers to a dietary pattern that primarily relies on grains as the main source of food. Compared to refined grains, a whole grain diet is rich in dietary fiber, vitamins, minerals, and antioxidants, which can improve blood glucose control, insulin sensitivity, and lipid levels. This dietary habit is associated with lower risks of cardiovascular diseases, diabetes, cancer, and mortality [3]. Energy restriction refers to limiting energy intake and is commonly used for weight control or weight loss. An energy-restricted diet can reduce body weight, fat content, and body fat percentage. This dietary habit can improve insulin sensitivity, reduce insulin resistance, lower the risk of cardiovascular diseases, and improve lipid levels [4]. Specific amino acid restriction refers to limiting the intake of specific amino acids, such as methionine, phenylalanine, etc. This type of diet can be used to treat certain genetic metabolic diseases, such as phenylketonuria [5]. Recent studies have shown that methionine restriction helps in improving neurological disorders and extending lifespan [6]. A diet with specific amino acid restriction can reduce the accumulation of pathological products, improve pathological symptoms, maintain amino acid balance in the body, and reduce the generation of harmful metabolic by-products.

Changes in dietary habits can affect the structure of the gut microbiota, and alterations in the gut microbiota can also regulate the development of metabolic diseases [7]. The gut microbiota is involved in the breakdown and metabolism of food, aiding in the digestion and utilization of nutrients. When the gut microbiota is imbalanced, the activity of digestive enzymes and other related enzymes may be inhibited, leading to the incomplete digestion and absorption of food. This can increase the risk of malnutrition and related diseases. Changes in the gut microbiota can also disrupt energy balance. Microbes can convert prebiotics in food into different metabolites, providing energy to intestinal cells and participating in the regulation of energy balance. This may result in excessive energy absorption and storage, thus increasing the risk of obesity and metabolic syndrome when the gut microbiota is imbalanced. In addition, an imbalanced gut microbiota can also affect toxin metabolism, leading to the accumulation of toxins in the body and adversely affecting metabolic health. Probiotics, prebiotics, and postbiotics are currently the most widely used methods to regulate the gut microbiota [8]. Probiotics can regulate the gut microbiota in various ways. Firstly, they occupy positions in the gut microbiota and competitively exclude harmful bacteria, maintaining the balance of the gut microbiota. Secondly, specific probiotics can produce beneficial metabolites, such as short-chain fatty acids, which help promote gut health and regulate energy metabolism. Research has also found that certain probiotics, such as *Bifidobacterium* and *Lactobacillus*, can improve metabolic health by regulating immune function, suppressing inflammatory responses, and enhancing the integrity of the intestinal mucosa [9]. Prebiotics are a type of carbohydrate that is not broken down by human digestive enzymes and can promote the growth and activity of beneficial bacteria. Prebiotics, such as inulin, oligofructose, and lactulose, are considered as the "food" for gut microbiota. Prebiotics improve the balance of the gut microbiota by providing nutrients that beneficial bacteria require for their growth. The intake of prebiotics can increase the abundance and diversity of beneficial bacteria, and regulate the gut ecosystem, thereby improving metabolic function and reducing the risk of metabolic diseases [10]. Postbiotics are important intermediates between the gut microbiota and human metabolism. Postbiotics help promote the diversity and balance of the gut microbiota, thereby providing protection against metabolic diseases [11]. Therefore, forming dietary habits that include an adequate supplementation of probiotics, prebiotics, and postbiotics contributes to metabolic health.

Furthermore, individualized dietary interventions for metabolic regulation in special populations are also necessary. Firstly, special populations may face unique health risks. For example, diabetes patients need to control blood glucose levels, hypertension patients need to limit sodium intake, and liver disease patients need to reduce fat intake. Personalized dietary interventions can develop suitable healthy eating plans according to the specific conditions of patients, forming personalized dietary habits, and reducing the risk of related diseases. Secondly, special populations may have specific nutritional needs. For instance, pregnant women need to increase the nutrients required during pregnancy, older adults need to consume adequate protein and vitamin D to maintain bone health, and athletes need to supplement enough protein and carbohydrates to improve sports performance [12]. Personalized dietary interventions can aid in developing specific dietary plans to meet the nutritional needs of different populations, providing the necessary nutrients for their bodies.

In conclusion, dietary habits are closely related to metabolic health. Therefore, it is crucial to explore the regulatory principles and mechanisms of different dietary habits on metabolic health, deepen our understanding of the relationship between dietary habits and health outcomes, promote healthy dietary habits, and prevent metabolic diseases.
